# Drama-based interventions for school oral health promotion among children and adolescents: a scoping review

**DOI:** 10.1186/s12903-026-07933-3

**Published:** 2026-02-19

**Authors:** Elena Shmarina, Johannes Todorov, Anida Saric

**Affiliations:** 1 Kalmar County Council, Public Dental Service, Oskarshamn, Sweden; 2 Centre for Oral Health, School of Health and Welfare, Jönköping University, Jönköping, Sweden; 3 Karlstad University, Karlstad, Sweden

**Keywords:** Health education, Behavioural change, Public health schools, School health services, Public health dentistry

## Abstract

**Background:**

Traditional school oral health education, delivered through lectures and printed materials, is often passive and overlooks emotional and behavioural learning. This highlights the need for more interactive approaches. Drama-based techniques, such as role-play and storytelling, engage children, foster reflection and encourage social interaction, though evidence for long-term oral health remains limited.

**Aim:**

To describe and summarise the available research on drama-based interventions among children and adolescents within school oral health promotion.

**Methods:**

This scoping review followed the Joanna Briggs Institute methodology and was reported according to the PRISMA-Scr criteria. The review protocol was prospectively developed and registered on the Open Science Framework (DOI: 10.17605/OSF.IO/CHQXE). A systematic search of six electronic databases (MEDLINE, CINAHL, DOSS, PsycInfo, Scopus and Web of Science) was conducted in November 2024 to identify original studies on the impact of drama-based interventions on children’s oral health. Grey literature sources were also searched to ensure broader coverage of relevant evidence. Titles and abstracts were independently screened by two reviewers, followed by full-text assessment for eligibility. From eligible studies, data were extracted to summarise, collate and make a narrative account of the findings.

**Results:**

This scoping review included 13 studies, selected from 14,955 records after screening, exclusions and adding reports from grey literature. Most were published within the past five years, reflecting rising interest in drama-based interventions within school oral health promotion. Studies showed global engagement but were predominantly conducted in Asia. Considerable variation in study design, intervention mode, duration and frequency were observed and most lacked theoretical grounding. Reported outcomes were largely positive for oral health-related knowledge, behaviours and hygiene, however evidence of clinical impact remained inconclusive. No studies involving adolescents could be identified, highlighting a clear gap in the evidence for this age group.

**Conclusions:**

Drama-based interventions in school oral health promotion suggest beneficial impact on improving children’s oral health knowledge, behaviours and hygiene, however evidence remains limited and long-term effects on caries prevention are unclear. Future research should address methodological weaknesses, integrate theory, include adolescents and expand across diverse cultural contexts to strengthen the evidence base.

**Supplementary Information:**

The online version contains supplementary material available at 10.1186/s12903-026-07933-3.

## Introduction

To ensure long-term oral health it is important to understand and adopt oral health practices from an early age, as a healthy lifestyle is more sustainable when established at a young age [1]. For this reason, school oral health promotion programmes can play a crucial role in fostering healthy lifestyles. These programmes have the advantage of reaching most school-aged children and adolescents and provide important networks to their families and communities [1].

Children’s and adolescents’ learning and behaviour development are complex processes influenced by cognitive, emotional and social development. Effective health promotion programmes need to address not only knowledge acquisition but also motivation and self-regulation, as these factors are crucial for sustainable behaviour change [2]. Despite this, conventional school-based oral health education often relies primarily on lectures, printed pamphlets and instructional videos, focusing on information delivery rather than fostering the skills and motivation needed for lasting behaviour change [3–5]. While these methods can provide essential knowledge, they often adopt a passive, one-way communication style that limits student engagement [6]. Furthermore, these approaches frequently fail to address the behavioural and emotional aspects of learning, which are crucial for lasting change. As a result, children may face challenges in retaining the information or applying it to their daily routines [6, 7]. This highlights the need for innovative and interactive intervention methods that go beyond traditional didactic teaching.

Approaches that adapt drama and theatre techniques, such as role-play, improvisation and storytelling, can create immersive learning experiences that actively engage students [6, 7]. By encouraging emotional connection, social interaction and reflective thinking, oral health promotion programmes utilising drama-based strategies have the potential to enhance oral health education and promote meaningful, lasting behaviour change among children [4].

Previous research suggests that drama-based interventions can positively influence children’s and adolescents’ knowledge and attitudes towards health behaviours across several areas of school health promotion, including mental, behavioural and social health [8, 9]. In the context of oral health, however, the application of drama-based approaches remains relatively underexplored. While some studies point to potential benefits, the current body of evidence is limited and inconsistent in terms of intervention design, delivery and evaluation [10–12]. This indicates the need for a comprehensive review of existing research to better understand the role and impact of drama-based interventions in school oral health promotion.

### Aim and objectives

The aim of this scoping review was to (i) describe and summarise the available research on drama-based interventions carried out amongst children and adolescents within school oral health promotion and (ii) identify existing knowledge gaps and directions for further research to advance policy and practice for school oral health promotion.

To address the aim, the objectives of this scoping review were to:

- Map the study designs and methods used.
- Describe the populations and school contexts involved.
- Identify the concepts, theories, or frameworks underpinning drama-based interventions.
- Summarise the reported impacts of drama-based interventions on child and adolescent oral health and associated factors.

### Methods

Based on the Population, Concept, and Context (PCC) framework (summarized in Table 1), the main research question guiding this review was: *What drama-based interventions have been used to promote oral health among children and adolescents in school settings?*

**Table 1:** Population, Concept, and Context (PCC) framework for the review

Population	Children and adolescents (school-aged population)
Concept	The core concept examined was drama-based interventions in school oral health promotion. For this review, drama-based intervention was conceptualised as an activity that promotes oral health through drama and theatre strategies (e.g. role-play, activating dialog, image work and theatre games), which are intended to foster students’ engagement in interactive, collaborative and affective learning, deepen their understanding of oral health and enable them to increase control over and to improve their oral health [7].
Context	The context for this review was school settings defined as settings providing learning space and learning environments for children and adolescents.

The review was conducted in accordance with the Joanna Briggs Institute (JBI) scoping review methodology [13] and the guidance of the PRISMA extension for scoping reviews (PRISMA-ScR) [14]. This methodology was chosen due to the heterogeneous and underexplored literature on drama-based interventions in school oral health and its ability to identify research gaps and guide future research [13, 15, 16]. The scoping review followed a multi-step, iterative process, including defining objectives and research questions, developing inclusion criteria, planning the review approach, searching and selecting evidence, extracting and analysing data and finally summarising findings in relation to the review question while noting implications [13, 16].

Prior to conducting this scoping review the Cochrane Library database for systematic reviews was searched for published or ongoing reviews on drama-based interventions within school oral health promotion. However, no review on the topic could be identified.

A scoping review protocol was developed and registered prospectively with the Open Science Framework on 21 December 2024 (Registration DOI: 10.17605/OSF.IO/CHQXE) [17].

### Eligibility criteria and sources of evidence

The inclusion and exclusion criteria for this scoping review were established to define the scope of eligible studies, as summarized below.

*Inclusion criteria:*

- Primary empirical studies (quantitative, qualitative, or mixed methods).
- Published in peer-reviewed journals or relevant grey literature (e.g., dissertations, theses, conference abstracts, pre-prints).
- Conducted in regular school settings with school-aged children and adolescents.
- English-language publications with accessible full texts.
- No time or geographic restrictions.

*Exclusion criteria:*

- Non-empirical studies (reviews, opinion pieces).
- Conducted in clinical or non-school community settings.
- Participants outside regular school contexts (e.g., children in care, with special needs).
- Use of non-drama creative arts (e.g., music, painting).
- Drama used solely as therapy (e.g., psychodrama) or only digital applications (e.g., video/computer games).
- Studies without oral health outcomes.

### Information sources and search strategy

The search strategy aimed to locate published studies and grey literature and was conducted in accordance with the JBI methodology [13]. It was developed, piloted, refined and executed with the collaboration of an experienced research librarian.

A preliminary search was conducted using MEDLINE (via PubMed) and Scopus (via Elsevier) to aid the evolution of the final search terms and to identify potential output of the scoping review. This search was followed by an analysis of text words contained in the title and an abstract of retrieved articles, as well as index terms used to describe the articles. Following consultation with the research team and the research librarian an initial set of search terms was systematically developed.

A secondary search was performed across all included databases to test the appropriateness of the selected terms. It was found that original terms captured a limited number of relevant publications. The search terms were revised, extended and re-trialled several times before consensus was achieved within the research team. The final search was performed on 19 November 2024 by the research librarian. The full search, as executed, is available in the appendix.

To identify potentially relevant literature the following bibliographic databases were searched: CINAHL with Full Text (EBSCOhost), Dentistry & Oral Science Source (EBSCOhost), MEDLINE (R) ALL (Ovid), PsycInfo (ProQuest), Scopus (Elsevier) and Web of Science Core Collection (Clarivate).

Scopus and Web of Science were chosen due to their extensive coverage of multidisciplinary literature. CINAHL and MEDLINE were also included as they represent core databases in the health sciences. Dentistry & Oral Science Source was selected due to its unique focus on oral health. In addition, PsycInfo was considered relevant due to its coverage of behavioural and social literature.

Moreover, searches were conducted using ProQuest Dissertations & Theses Global (ProQuest) and Google Scholar. For these platforms the search strategy was modified by removing MeSH and TIAB field tags, retaining only the original keywords. This adjustment was necessary because grey literature, including dissertations and theses, often features non-standardised terminology and is not consistently indexed using controlled vocabularies [18]. Further, Google Scholar does not support MeSH or field-specific syntax. Therefore, employing a simplified search with broad keywords increases the likelihood of capturing relevant materials in grey literature sources. To manage the results in Google Scholar the first 20 pages was screened for suitability [19]. The reference list of all included publications as well as citations was screened for additional studies.

Detailed search strategies for each database are provided in the Appendix.

### Study selection

Following the final search, all records were collated and de-duplicated by the research librarian using Deduplicator (https://tera-tools.com/) before being imported into the screening software Rayyan (http://rayyan.qcri.org), designed to facilitate screening and study selection.

**Fig. 1:**
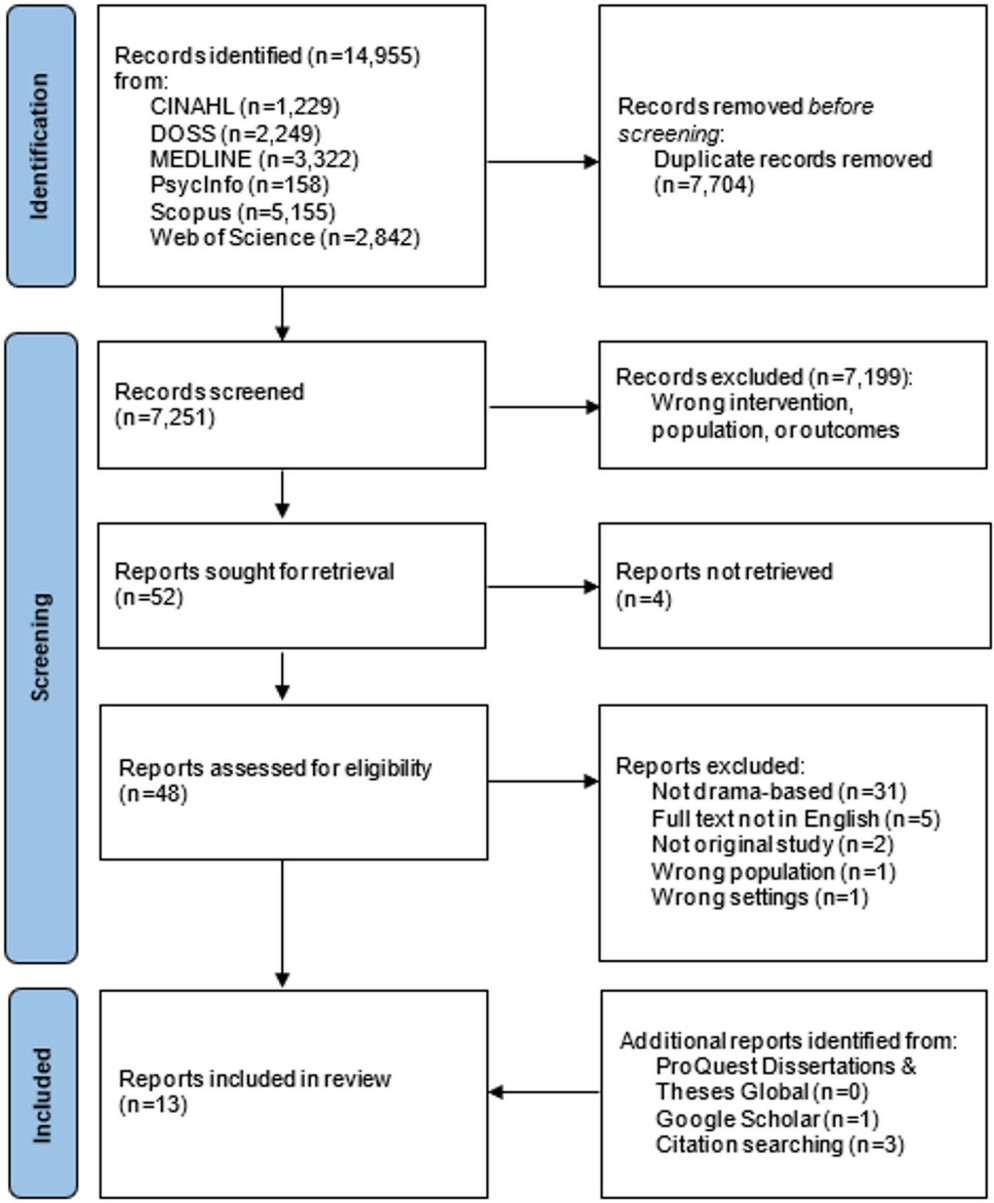
Flow diagram for scoping review

For calibration, two reviewers (ES and JT) independently screened the same 20 citations, selected from the search results, discussed discrepancies and refined the guidance before starting with the screening for this review. Titles and abstracts, and thereafter articles in full text, were independently screened by the same two reviewers (ES and JT) for assessment against the eligibility criteria. Any disagreements that arose at each stage of the selection process were solved in discussion between all three reviewers (ES, JT and AS) until consensus was reached. The results of the search, the inclusion process and reasons for exclusion of full-text articles are presented in a PRISMA flow chart (Fig. 1).

An assessment of the methodological quality of the reviewed studies was not performed, as the purpose of a scoping review is to provide an overview of the scope and characteristics of the available evidence, rather than to provide clinically conclusive answers [13].

### Charting the data

A data-extraction form, accompanied by detailed guidance, was developed and piloted prior to the commencement of the review. The extracted information included first author, year of publication, country of origin (i.e. where the study was conducted), study objectives, participants’ demographics, settings, methodology, types of drama-based strategies, outcome measures and key findings. Data extraction was completed by two reviewers (ES and JT) and verified by a third reviewer (AS). In case of inconsistency, all three reviewers were involved in the discussion to ensure that all relevant data items were extracted. When additional data were required authors of the original studies were contacted, though responses were not obtained in all cases.

### Summarising and reporting the findings

The characteristics of interest were collated and charted into a Microsoft Excel database. Given the wide range of outcomes reported, results were grouped into three domains to facilitate analysis: *psycho-behavioural*,* clinical* and *self-reported*. This categorisation reflects variations in both the levels of impact and the outcome measures applied. The findings are presented in tabular form and accompanied by narrative summary.

## Results

### Study selection

A total of 14,955 records were identified through electronic databases. After removing 7,704 duplicates, the titles and abstracts of the remaining 7,251 records were screened for eligibility. Full texts of 52 reports were assessed; 4 could not be retrieved and 40 were excluded, with reasons for exclusion detailed in Fig. 1. Searches of grey literature and citations yielded an additional 4 reports, resulting in a total of 13 reports included in this scoping review. Study selection is summarized in Fig. 1.

### General characteristics of reviewed studies

The general characteristics of the reviewed studies are outlined in Table 2 to provide an overview of the extent, range and nature of the evidence in the area.

**Table 2:** General characteristics

Characteristic	Number of studies	Reference
Year of publication		
2000–2009	1	[20]
2010–2019	4	[21–24]
2020 and after	8	[25–32]
Country		
India	6	[21, 23–26, 28]
Turkey	1	[27]
Indonesia	2	[30, 31]
Malaysia	1	[32]
Germany	1	[20]
Peru	1	[29]
Brazil	1	[22]
Study design		
Experimental (RCT)	3	[21, 23, 27]
Quasi-experimental	5	[20, 22, 25, 29, 30]
Pre-experimental	5	[24, 26, 28, 31, 32]
Population		
Pre-school children	9	[20–23, 27–30, 32]
School children	4	[24–26, 31]
Adolescents	0	
Settings		
Primary/Kindergarten	5	[22, 27, 28, 30, 32]
Secondary	3	[21, 23, 24]
General/unspecified	5	[20, 25, 26, 29, 31]

The publication years of the reviewed studies spanned more than two decades, with one study being published between 2000 and 2009 [20], four between 2010 and 2019 [21–24] and the majority (*n* = 8) from 2020 onwards [25–32]. The studies were conducted across a range of countries, with India contributing the highest number (*n* = 6) [21, 23–26]. The remaining studies were conducted in Indonesia (*n* = 2) [30, 31], Malaysia (*n* = 1) [32], Turkey (*n* = 1) [27], Peru (*n* = 1) [29], Brazil (*n* = 1) [22] and Germany (*n* = 1) [20]. Methodologically, all relied on quantitative approaches: three randomized controlled trials [21, 23, 27], five quasi-experimental studies [20, 22, 25, 29, 30] and five pre-experimental studies [24, 26, 28, 31, 32]. No qualitative or mixed-method studies were identified.

Sample sizes ranged from 44 to 360 participants, with most studies including between 100 and 180 participants (*n* = 6) [20, 21, 23, 24, 27, 29]. Less than half of the studies reported using a power calculation to determine sample size (*n* = 6) [22, 23, 25–27, 29], while the remaining studies did not specify how their sample sizes were established (*n* = 7) [20, 21, 24, 28, 30–32].

The reviewed studies primarily targeted pre-school children, with the majority (*n* = 9) focusing on this age group [20–23, 27–30, 32]. The remaining four studies [24–26, 31] involved school-aged children. No studies involving adolescents could be identified, highlighting a clear gap in the evidence for this age group.

The studies were conducted across a variety of school settings, most frequently pre-schools and kindergartens (*n* = 5) [22, 27, 28, 30, 32], followed by secondary schools, high schools (*n* = 2) [21, 23] and a senior school (*n* = 1) [24]. In the remaining five studies school was classified as general or unspecified in level, such as private (*n* = 2) [25, 26], public (*n* = 2) [29, 31] or regular schools (*n* = 1) [20]. Terminology describing school type varied across the included studies, and in some cases, schools were referred to as higher secondary despite participants being predominantly of primary or elementary school age. Detailed descriptions of school structure were not consistently reported.

### Theoretical grounding

The theoretical underpinning for interventions were largely absent in the reviewed studies. An exception was identified in one study [28] which situated its storytelling intervention within Fogg’s behaviour model [33], a framework that emphasises the interplay of motivation, ability and appropriate triggers as determinants of behavioural change. Similarly, hypotheses were rarely reported, with just three studies [22, 25, 26] providing clear formulations, while most others omitted them.

### Characteristics of drama-based interventions

Drama-based interventions demonstrated considerable heterogeneity in mode, duration and frequency, complicating cross-study comparisons (Table 3). Among the reviewed studies drama performed by facilitators (*n* = 5) [21, 23, 25, 26, 30] or using puppets (*n* = 5) [20, 24, 29, 31, 32] were most frequently implemented, followed by playful learning (*n* = 2) [22, 27] and storytelling formats (*n* = 1) [28].

**Table 3:** Characteristics of drama-based interventions

First author (year)	Drama-based intervention mode	Drama-based intervention parameters			
		Session duration	Session frequency	Intervention length	Evaluation time
Akkaya (2021)	Playful learning interventions with toys, and visual and auditory sources including children’s engagement in discussion.	65 min	Once a week	5 weeks	7 weeks
Chandran (2019)	Videographed drama enacted by senior students at the school.	15 min	Once	NA	3 months
GeethaPriya (2020a)	Drama using scenes and dialogues was enacted by dental residents dressed as cartoon characters.	20 min	Every 3 or 6 months	2 years	2 years
GeethaPriya (2020b)	Drama using script and dialogues was enacted by dental residents dressed as cartoon characters.	20 min	Every 6 months	2 years	2 years
John (2013)	Drama enacted by the senior dental residents trained by dentist, disguised as cartoon characters.	20 min	Once	NA	3 months
Kaur (2014)	Puppet show regarding oral health.	NR	Daily	4 days	1 week
Ladera Castaneda (2022)	Puppet theatre sessions ending with 5 min of feedback where the puppets interacted with the children.	30 min	Once a week the first month and then every second week	4 months	4 weeks 4 months
Makuch (2001)	Puppet show in combination with games, such as guided role-play, food matching game, error story and toothbrushing song.	NR	5 times a week	5 weeks	5 weeks 10 weeks
Putri (2024)	Dental puppet counselling conducted in an interactive manner, using a role-playing activity and discussion and focusing on proper toothbrushing techniques and the importance of maintaining oral hygiene.	30 min	Once	NA	Immediately
Rahina (2022)	Drama story with the theme of how to promote oral health using popular character in cartoon film performed by dental student.	NR	Every 6 months	2 years	2 years
Seah (2025)	A live puppet show featuring five puppet characters focusing on the appropriate brushing frequency and healthy foods performed on day 1, followed by daily screenings of a video recording of the show.	NR	Daily	2 weeks	Day 1 Day 7 Day 14
Shruti (2021)	Storytelling containing the oral health messages delivered using hand puppets.	15–20 min	Once	NA	1 week
Sigaud (2017)	Playful learning intervention with a giant mouth model, toys, visual and auditory sources including conversation circles conducted with children.	60 min	3 sessions	1 to 4 days between each session	3 to 7 days after the third session

NR – Not Reported

NA – Not Applicable

Most studies favoured short sessions of 15–30 min (*n* = 7) [21, 23, 25, 26, 28, 29, 31], which may reflect practical considerations for maintaining engagement in young children, though a minority used longer sessions (*n* = 2) [22, 27] or did not report duration (*n* = 4) [20, 24, 30, 32]. Session frequency and intervention length varied widely, ranging from single sessions with immediate or short-term follow-up [21, 23, 27, 28, 31] to multi-week or multi-year programmes [20, 22, 24–26, 29, 30, 32], highlighting the diversity of study designs.

### Outcome domains of drama-based interventions

**Fig. 2:**
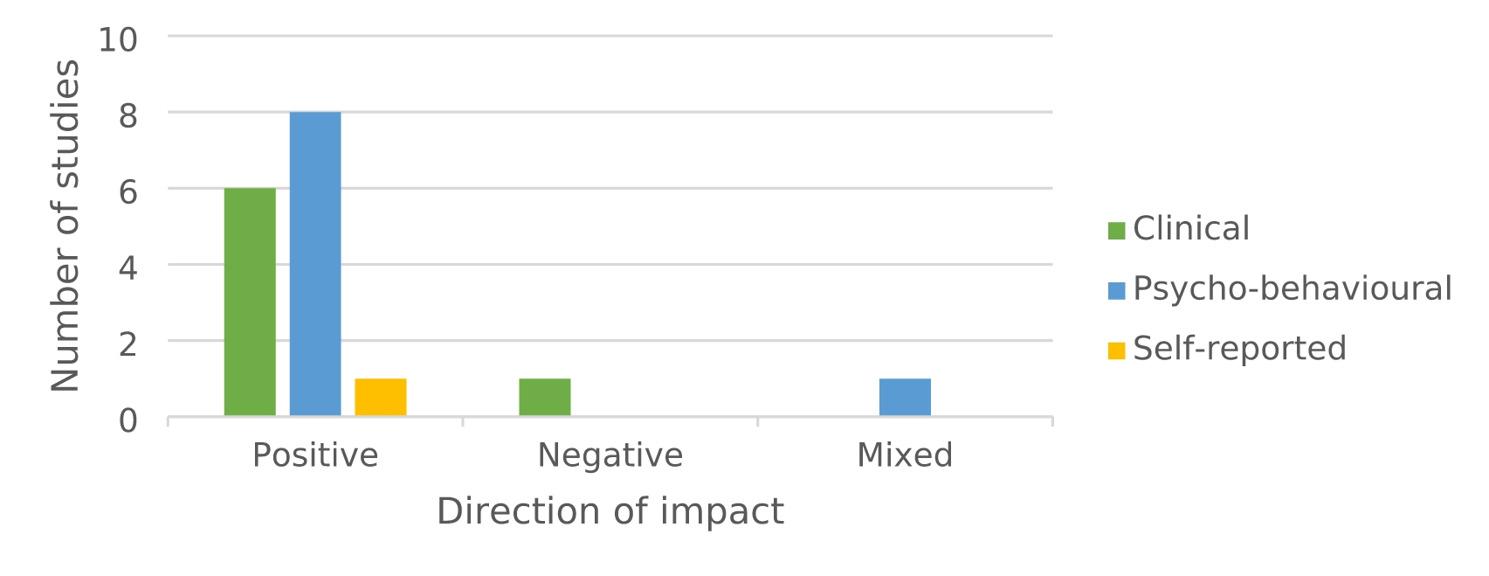
Number of studies demonstrating impact direction across outcome domains

Most of the reviewed studies reported that drama-based interventions were effective in improving children’s oral health-related knowledge, attitudes and practices, as well as their overall oral health status. The studies, however, assessed a wide variety of outcomes using different tools and approaches, which made direct comparisons challenging. To provide a coherent synthesis the results were grouped into three domains, *psycho-behavioural*,* clinical* and *self-reported*, reflecting both the different levels at which interventions had an impact and the diversity of measures used. Outcome domains with their associated outcomes, reported impact and its direction are presented in Table 4; Fig. 2.

**Table 4:** Summary of main findings across outcome domains of drama-based interventions

First author (year)	Study location	Study design	Participants (n, age)	Settings^a^	Intervention (participants n)	Outcome domain (oral health-related outcome)	Measurement tool (item^b^)	Reported impact	Direction of impact
Akkaya (2021)	Turkey	Experimental (RTC)	n = 100, 4–6 years	Kindergarten	Playful learning (n = 50) vs. control (n = 50)	Psycho-behavioural (toothbrushing behaviour)	Toothbrushing protocol (7-item)	Improved toothbrushing behaviour	Positive
						Clinical (oral hygiene)	S&L PI	Decreased amount of plaque	Positive
Chandran (2019)	India	Experimental (RTC)	n = 120, 3–5 years	Aided higher secondary school	Videographed drama (n = 40) vs. OHE by dentists (n = 40) vs. control (n = 40)	Clinical (oral hygiene)	DI-S	Improved oral hygiene; drama most effective	Positive
GeethaPriya (2020a)	India	Quasi-experimental	n = 360, 8–9 years	Private school	Drama (n = 109) vs. board game (n = 133) vs. flashcards (n = 118)	Clinical (oral hygiene)	OHI-S	Improved oral hygiene; drama and game more effective	Positive
						Clinical (caries experience)	dft-index	Improved oral health status in all groups	Positive
						Clinical (caries experience)	DMFT-index	Increased caries prevalence in all groups; drama highest	Negative
						Clinical (caries treatment needs)	CTN primary	Reduced caries treatment needs in all groups	Positive
						Clinical (caries treatment needs)	CTN permanent	Increased caries treatment needs in drama and flashcards groups	Negative
						Self-reported (OHRQoL)	COHIP-SF (19-item)	Improved OHRQoL for all groups	Positive
GeethaPriya (2020b)	India	Pre-experimental	n = 360, 8–9 years	Private school	Drama (n = 109) vs. board game (n = 133) vs. flashcards (n = 118)	Psycho-behavioural (oral health related knowledge, attitude and practice)	KAP (18-item)	Increased oral health-related knowledge for all groups	Positive
John (2013)	India	Experimental (RTC)	n = 100, 4–6 years	Higher secondary school	Drama (n = 24) vs. OHE by dentist (n = 25) vs. OHE by teacher (n = 28) vs. control (n = 23)	Clinical (oral hygiene)	DI-S	Improved oral hygiene; drama most effective	Positive
Kaur (2014)	India	Pre-experimental	n = 100, 5 years and older^c^	Senior secondary school	Puppet show (n = 100)	Psycho-behavioural (oral health knowledge)	Questionnaire (20-item)	Increased oral health knowledge	Positive
						Clinical (oral health status)	Observational checklist (5-item)^d^	Improved oral health status	Positive
Ladera Castaneda (2022)	Peru	Quasi-experimental	n = 132, 3–5 years	Public school	Puppet theatre: 3 years (n = 44) vs. 4 years (n = 44) vs. 5 years (n = 44)	Psycho-behavioural (oral health knowledge)	Questionnaire (5-item)	Increased oral health knowledge in all groups for urban children but not for rural	Mixed
						Clinical (oral hygiene)	DI-S^e^	Improved oral hygiene	Positive
Makuch (2001)	Germany	Quasi-experimental	n = 180, 3–5 years	Regular school	Younger/older age (n = 90 per group): puppet show + games (n = 30) vs. games + puppet show (n = 30) vs. control (n = 30)	Psycho-behavioural (oral hygiene knowledge and skills)	Semi-structured interview + observation by trained raters (NR)	Improved oral hygiene knowledge and skills for all experimental groups	Positive
Putri (2024)	Indonesia	Pre-experimental	n = 52, NR^f^	Public elementary school	Dental puppet counselling (n = 52)	Psycho-behavioural (oral health maintenance knowledge)	Questionnaire (15-item)	Increased oral health maintenance knowledge	Positive
Rahina (2022)	Indonesia	Quasi-experimental	n = 240, 4–6 years	Kindergarten school	Drama (n = 80) vs. drama and toothbrushing (n = 80) vs. control (n = 80)	Clinical (caries experience)	dmft-index	Reduced caries prevalence; toothbrushing provides no added benefit	Positive
Seah (2025)	Malaysia	Pre-experimental	n = 51, 4–6 years	Pre-school	Puppet show (n = 51)	Psycho-behavioural (oral health knowledge)	Questionnaire (5-item)	Increased and sustained oral health knowledge	Positive
Shruti (2021)	India	Pre-experimental	n = 220, 3–6 years	Pre-school	Storytelling (n = 220)	Psycho-behavioural (oral health related knowledge, attitude and practice)	KAP (18-item)	Improved oral health related knowledge, attitude and practice	Positive
Sigaud (2017)	Brazil	Quasi-experimental	n = 44, 3–5 years	Pre-school	Playful learning: 3 years (n = 14) vs. 4 years (n = 12) vs. 5 years (n = 8)	Psycho-behavioural (toothbrushing behaviour)	Systematized observation using structured protocol (10-item)	Increase in the number of appropriate toothbrushing behaviours	Positive

*COHIP-SF* Child Oral Health Impact Profile, *COHIP-SF* Child Oral Health Impact Profile, *DI-S* Debris Index Simplified (component of the OHI-S), *KAP* Oral Health-related Knowledge, Attitude and Practice, *NR* Not Reported, *OHE* Oral Health Education, *OHI-S* Oral Hygiene Index Simplified, *OHRQoL* Oral Health-Related Quality of Life, *RTC* Randomised Control Trial, S&L PI Silness & Löe Plaque Index

^a^ Age-school mismatch due to insufficient reporting of school type and structure,^b^ For non-clinical measurement tools, ^c^ Primary school children (70% of participants are 5–10 years old and 30% are 10 years and above), ^d^ Oral health status measured via checklist (foul smell, lips, tongue, gums and teeth), ^e^ Referred as BPI (Bacterial Plaque Index) in Ladera Castaned (2022),^f^ Second grade students (age is not reported)

### Psycho-behavioural outcome domain

The psycho-behavioural outcome domain encompasses children’s oral health-related knowledge, attitudes and practices (KAP), including their understanding of oral hygiene, motivation to maintain healthy routines and actual toothbrushing habits. Within this domain, outcomes were primarily measured using questionnaires completed by children, with toothbrushing practices further evaluated through structured observations conducted by researchers, trained raters or parents. These outcomes were explored in nine studies [20, 22, 24, 26–29, 31, 32], the majority of which reported consistent improvements among pre-school and early school-aged children following the implementation of drama-based interventions.

Five studies investigated drama-based interventions using puppets, with all reporting positive effects on children’s oral health knowledge and hygiene skills. For example, Makuch et al. [20] reported significant improvements in experimental groups compared to controls, highlighting the advantage of developmentally-tailored playful methods over didactic instruction. Similarly, Ladera Castaneda et al. [29] found that puppet theatre increased knowledge among urban pre-schoolers, although children from rural areas did not show significant gains. Other studies incorporating puppets in their interventions similarly reported positive outcomes. Kaur et al. [24] observed significant increases in knowledge scores in children aged five years and older; Putri et al. [31] found enhanced oral health maintenance knowledge in primary school children; and Seah et al. [32] reported both immediate and sustained improvements in oral health knowledge among pre-schoolers aged 4–6 years.

Additionally, two studies examined playful learning on toothbrushing behaviour, both reporting significant improvements: Akkaya et al. [27] between intervention and control groups and Sigaud et al. [22] pre- to post-intervention. Another two studies examined the effects of creative interventions on children’s oral health-related KAP, both reporting significant improvements. Shruti et al. [28] used storytelling in pre-schoolers, significantly increasing mean KAP scores while GeethaPriya et al. [26] employed drama, flashcards and board games, finding significant gains across all modes with no differences between them.

### Clinical outcome domain

The clinical outcome domain encompasses objective measures of children’s oral health, including oral hygiene levels, dental caries and caries treatment needs (CTN) and were conducted by trained clinicians or examiners. These outcomes were examined across seven studies [21, 23–25, 27, 29, 30], with results showing occasional inconsistencies.

Six studies assessed oral hygiene and oral health status, with all reporting improvements. Two randomised controlled trials [21, 23] found that drama interventions produced significantly greater improvements in oral hygiene compared with conventional didactic education delivered by dentists or teachers, as well as in comparison to a control group. GeethaPriya et al. [25] compared drama, game and flashcards over two years and found that all three improved oral health status, with drama and game outperforming flashcards. Two other studies reported significant plaque reductions following playful learning [27] and puppet-theatre [29] interventions, although the latter found no significant effect among rural pre-schoolers compared with urban children [29]. Kaur et al. [24] also found that a puppet show significantly improved oral health status, as measured by a checklist.

Dental caries was evaluated in two studies, showing some inconsistency in the results. Rahina et al. [30] reported that biannual drama in 4–6-year-olds effectively prevented caries, with no additional benefit from combining drama with toothbrushing. In contrast, GeethaPriya et al. [25] found that DMFT scores increased for drama, game and flashcard interventions, most notably for drama, whereas dft scores decreased across all groups, reflecting a mixed impact on caries measures. The study also assessed CTN, showing a significant reduction in treatment needs for primary teeth across all groups, while permanent teeth had significantly increased treatment needs in the drama and flashcard groups [25].

### Self-reported outcome domain

The self-reported outcome domain reflects children’s perceptions of their oral health and its impact on daily life. Only one study assessed school children’s OHRQoL using the COHIP tool [25]. The study reported significant increases in total COHIP scores over two years for drama, game and flashcards interventions, with the highest scores observed in the drama group.

## Discussion

This scoping review sought to provide a comprehensive overview of existing research on the impact of drama-based interventions on oral health and related outcomes among children and adolescents. Despite the limited number of identified studies and the heterogeneity in study characteristics, which restricts broader generalisation, some notable observations can still be made.

While the results do not support definitive conclusions, they suggest that drama-based interventions in school settings can be effective in improving children’s oral health-related knowledge, attitudes, behaviours and clinical outcomes. These findings align with evidence from other health fields where the research base is also relatively scarce. Nonetheless, interventions grounded in a range of creative activities, including drama, have been shown to positively influence children’s confidence, resilience, mental health, health-related behaviours and wellbeing [34–37]. Thus our results, together with existing research from the broader health context, highlight the growing potential of creative strategies to support children’s health, yet the evidence base in both general and oral health remains limited and fragmented.

Furthermore, in order to provide a coherent synthesis the findings were grouped into three outcome domains: *psycho*-*behavioural*, *clinical* and *self-reported*. These domains are connected and can be seen as a natural pathway through which drama-based interventions exert their impact. Initially these interventions target children’s knowledge, attitudes and behaviours by providing active, meaningful engagement. As these behavioural changes accumulate they are likely to contribute to improvements in clinical outcomes, reducing risk factors for oral disease. Ultimately the combined influence of healthier behaviours and better clinical status shapes how children perceive and self-report their oral health and overall wellbeing. This way of understanding helps to explain both the diversity of outcomes reported in the reviewed studies and the multi-level effects of drama-based approaches. Consequently, framing our results in this way offers a clearer view of how the evidence on this topic has developed. The findings suggest a gradient in both outcomes and the development of research on drama-based interventions. Psycho-behavioural outcomes are consistently positive, demonstrating the interventions’ effectiveness in shaping children’s knowledge, attitudes and behaviours. Clinical outcomes are more variable, reflecting the ongoing challenges of translating behavioural improvements into measurable oral health gains. Self-reported outcomes are rarely studied, but the limited evidence points to beneficial effects, highlighting an underexplored area. Thus, this pattern indicates that the field is still emerging: early studies establish feasibility and proximal effects, clinical impacts are less predictable and subjective experiences remain largely unexamined [38].

Additionally, the findings reveal a limited engagement of the research community on drama-based interventions within school oral health promotion, as only a small number of studies from a broad search met the inclusion criteria and were reviewed. However, most of the studies were published in the past five years, highlighting growing scholarly interest in the field. This trend can suggest increasing recognition of creative, participatory methods as engaging tools for oral healtha education, that emphasise learning through social interaction and active engagement [39]. These approaches provide opportunities for children to co-construct knowledge, practice skills in context and learn collaboratively, supporting the development of oral health-related skills and behaviours [7].

### Strengths and limitations

A key strength of this scoping review is its systematic approach to mapping relevant studies according to the mode of intervention. However, the inconsistency in how drama-based strategies are defined and applied across the reviewed studies posed challenges for direct comparison and synthesis of data. Despite this, the use of a defined protocol, structured data extraction procedures and regular team consultations ensured analytical rigor and consistency in reporting [13].

Furthermore, the screening and data extraction processes were conducted independently by two reviewers and verified by the third reviewer, which helped minimise the risk of bias. The use of several electronic databases, combined with grey literature searches and citation tracking, contributed to a robust and inclusive evidence-gathering process. This comprehensive strategy increased the reliability and depth of the review. However, the inclusion of numerous databases generated a substantial number of duplicate records due to the overlap between databases.

Additionally, some articles could not be retrieved in full text and were therefore excluded, potentially limiting the comprehensiveness of the review. The review was limited to studies published in English, possibly excluding significant findings from non-English sources. In four instances specific key details were missing from the included articles. Although corresponding authors were contacted to obtain the missing information, none of them responded, which may have affected the thoroughness of the data synthesis.

### Gaps in the research

This review revealed several important gaps in research on drama-based interventions within school oral health promotion, including methodological limitations, underrepresentation of diverse populations and geographical concentration.

The number of identified studies was limited and the existing literature is predominantly quantitative, with a noticeable lack of qualitative research. The variation in intervention types and delivery methods further complicates comparisons across studies. The long-term effects of drama-based interventions remain underexplored. Most studies focused on immediate or short-term outcomes, providing little insight into sustained oral health-related changes over time. Moreover, only few studies utilised randomised controlled trial designs, while most adopted quasi-experimental or pre-experimental approaches, frequently without control groups or randomisation. This reliance on non-randomised and observational study designs reduces the strength of causal inferences that can be drawn about the effectiveness of drama-based interventions [40]. Compounding this issue, many studies lacked clear theoretical frameworks and explicitly-stated hypotheses, which limits understanding of how drama-based interventions influence oral health outcomes [41]. This omission may weaken the explanatory power of findings, hinder the development of effective interventions and limit the accumulation of transferable knowledge across studies [42].

Furthermore, the child’s perspective is largely absent from the literature, which is critical when evaluating interventions aimed at children. This restricts the ability to explore children’s subjective experiences and deeper insights into how and why such interventions may be effective.

A notable population gap was observed, with most studies targeting pre-school-aged children and only a few including those in primary school. No studies involving adolescents were found, despite evidence from other health-related fields indicating that drama-based interventions are effective with this age group [8].

Geographically, the current body of evidence is concentrated in Asia. While these studies provide valuable insights, there is a clear need for research in other global regions, where oral health systems, school settings and cultural attitudes towards health education may differ significantly.

### Implications for research

To strengthen future research in this area, greater consistency in intervention design and reporting is essential to enable meaningful comparisons and potential meta-analyses. Integrating theoretical frameworks and clearly defined hypotheses would also enhance the design and interpretation of findings. Moreover, future studies should prioritise longitudinal and randomised controlled trials to assess both the short- and long-term effectiveness of interventions. There is also a need to increase the number of qualitative and mixed-methods studies to gain a deeper understanding of how drama-based interventions work and to explore contextual and process-related factors that may influence their effectiveness. Including the voices and perspectives of both children and their caregivers is particularly important to ensure interventions are relevant, engaging and appropriately tailored. Finally, expanding research efforts to a wider range of global regions is crucial to examine the transferability and effectiveness of these interventions across different cultural settings.

## Conclusion

Research on school-based drama interventions is still scarce, with considerable variation in how interventions are designed and assessed. Despite these limitations, such interventions have been found to improve children’s oral health-related knowledge, behaviours and hygiene, often outperforming the effectiveness of traditional teaching methods. However, their long-term impact on caries prevention remains uncertain, underscoring the need for well-designed longitudinal studies. This review also highlights significant research gaps, including methodological limitations, limited theoretical grounding, underrepresentation of adolescents and a concentration of studies in certain regions. Addressing these gaps through more rigorous, theory-informed and culturally diverse research will be essential for developing a stronger and more generalisable evidence base in this field.

## Supplementary Information

Supplementary Material 1.

## Data Availability

The datasets supporting the conclusions of this article are included within the article.

## Abbreviations

CTN: Caries treatment needs
COHIP: Child oral health impact profile
DMFT: Decayed missing filled teeth
dft: Decayed filled teeth
KAP: Oral health–related knowledge, attitude and practice
OHRQoL: Oral health–related quality of life
PRISMA-Scr: Preferred reporting items for systematic reviews and meta–analyses extension for scoping reviews
