# Supplementary material for: Drama-based interventions for school oral health promotion among children and adolescents: a scoping review
Source: BMC Oral Health. 2026 Feb 19;26:522. doi: 10.1186/s12903-026-07933-3 (PMC13020286; doi:10.1186/s12903-026-07933-3)
Supplement: Supplementary file 1 — Supplementary Material 1. [file 12903_2026_7933_MOESM1_ESM.docx]

# Characteristics and impact of drama-based interventions carried out amongst children and adolescents within school oral health promotion

Search strategy protocol

**Databases searched**CINAHL with Full Text (EBSCOhost) n= **1,229**
Date of search: November 19, 2024
Search mode: Advanced Search

|  | **Query** | **Results** |
| --- | --- | --- |
| #1 | TI ( drama* OR game* OR play OR plays OR puppetry OR theater* OR theatre* OR storytelling OR “guided imagery” OR improvisation OR pantomime ) OR AB ( drama* OR game* OR play OR plays OR puppetry OR theater* OR theatre* OR storytelling OR “guided imagery” OR improvisation OR pantomime ) OR SU ( drama* OR game* OR play OR plays OR puppetry OR theater* OR theatre* OR storytelling OR “guided imagery” OR improvisation OR pantomime ) | 227,222 |
| #2 | MH "Drama" OR MH "Guided Imagery" OR MH "Performing Arts" OR MH "Play and Playthings" OR MH "Storytelling" | 20,277 |
| #3 | #1 OR #2 | 229,100 |
| #4 | TI ( “dental caries” OR “dental cavity” OR “dental health” OR “dental hygiene” OR ”DMF index” OR ”mouth health” OR ”mouth hygiene” OR “oral health” OR “oral hygiene” OR toothbrush* OR “tooth health” ) OR AB ( “dental caries” OR “dental cavity” OR “dental health” OR “dental hygiene” OR ”DMF index” OR ”mouth health” OR ”mouth hygiene” OR “oral health” OR “oral hygiene” OR toothbrush* OR “tooth health” ) OR SU ( “dental caries” OR “dental cavity” OR “dental health” OR “dental hygiene” OR ”DMF index” OR ”mouth health” OR ”mouth hygiene” OR “oral health” OR “oral hygiene” OR toothbrush* OR “tooth health” ) | 51,145 |
| #5 | MH "Oral Health" OR MH "Mouth Care+" | 24,083 |
| #6 | #4 OR #5 | 51,801 |
| #7 | #3 AND #6 | 1,229 |

Controlled terms comment: Mouth Care+ includes Oral Hygiene, Toothbrushing

Dentistry & Oral Sciences Source (EBSCOhost) n=2,249
Date of search: November 19, 2024
Search mode: Advanced Search

|  | **Query** | **Results** |
| --- | --- | --- |
| #1 | TI ( drama* OR game* OR play OR plays OR puppetry OR theater* OR theatre* OR storytelling OR “guided imagery” OR improvisation OR pantomime ) OR AB ( drama* OR game* OR play OR plays OR puppetry OR theater* OR theatre* OR storytelling OR “guided imagery” OR improvisation OR pantomime ) OR KW ( drama* OR game* OR play OR plays OR puppetry OR theater* OR theatre* OR storytelling OR “guided imagery” OR improvisation OR pantomime ) | 13,692 |
| #2 | DE "CHILDREN'S plays" OR DE "CHILDREN'S theater" OR DE "DRAMA" OR DE "GAMES" OR DE "PLAY" OR DE "PERFORMING arts" OR DE "PUPPET plays" OR DE "PUPPET theater" OR DE "STORYTELLING" OR DE "THEATER" | 117 |
| #3 | #1 OR #2 | 13,748 |
| #4 | TI ( “dental caries” OR “dental cavity” OR “dental health” OR “dental hygiene” OR ”DMF index” OR ”mouth health” OR ”mouth hygiene” OR “oral health” OR “oral hygiene” OR toothbrush* OR “tooth health” ) OR AB ( “dental caries” OR “dental cavity” OR “dental health” OR “dental hygiene” OR ”DMF index” OR ”mouth health” OR ”mouth hygiene” OR “oral health” OR “oral hygiene” OR toothbrush* OR “tooth health” ) OR KW ( “dental caries” OR “dental cavity” OR “dental health” OR “dental hygiene” OR ”DMF index” OR ”mouth health” OR ”mouth hygiene” OR “oral health” OR “oral hygiene” OR toothbrush* OR “tooth health” ) | 55,434 |
| #5 | DE "TOOTH care & hygiene" OR DE "DENTAL care" OR DE "ORAL hygiene" OR DE "CAVITY prevention" OR DE "DENTAL hygiene" OR DE "TEETH polishing" | 53,614 |
| #6 | #4 OR #5 | 86,517 |
| #7 | #3 AND #6 | 2,249 |

MEDLINE (R) ALL (Ovid) n=3,322
Date of search: November 19, 2024

|  | **Query** | **Results** |
| --- | --- | --- |
| #1 | (drama* or game* or play or plays or puppetry or theater* or theatre* or storytelling or "guided imagery" or improvisation or pantomime).tw,kf. | 1,723,200 |
| #2 | Drama/ or Imagery, Psychotherapy/ or "Play and Playthings"/ | 14,029 |
| #3 | #1 OR #2 | 1,730,797 |
| #4 | ("dental caries" or "dental cavity" or "dental health" or "dental hygiene" or "DMF index" or "mouth health" or "mouth hygiene" or "oral health" or "oral hygiene" or toothbrush* or "tooth health").tw,kf. | 84,441 |
| #5 | DMF Index/ or Oral Health/ or Oral Hygiene Index/ or Oral Hygiene/ or Toothbrushing/ | 46,548 |
| #6 | #4 OR #5 | 98,165 |
| #7 | #3 AND #6 | 3,322 |

Controlled terms comment: Imagery, Psychotherapy is used for Guided imagery

PsycInfo (ProQuest) n=158
Date of search: November 19, 2024
Search Mode: Advanced search

|  | **Query** | **Results** |
| --- | --- | --- |
| #1 | tiab(drama* OR game* OR play OR plays OR puppetry OR theater* OR theatre* OR storytelling OR “guided imagery” OR improvisation OR pantomime ) OR subject(drama* OR game* OR play OR plays OR puppetry OR theater* OR theatre* OR storytelling OR “guided imagery” OR improvisation OR pantomime ) | 305,075 |
| #2 | MAINSUBJECT.EXACT("Drama") OR MAINSUBJECT.EXACT("Guided Imagery") OR MAINSUBJECT.EXACT("Improvisation") OR MAINSUBJECT.EXACT("Storytelling") OR MAINSUBJECT.EXACT("Theatre") | 12,278 |
| #3 | #1 OR #2 | 305,075 |
| #4 | tiab(“dental caries” OR “dental cavity” OR “dental health” OR “dental hygiene” OR ”DMF index” OR ”mouth health” OR ”mouth hygiene” OR “oral health” OR “oral hygiene” OR toothbrush* OR “tooth health”) OR subject(“dental caries” OR “dental cavity” OR “dental health” OR “dental hygiene” OR ”DMF index” OR ”mouth health” OR ”mouth hygiene” OR “oral health” OR “oral hygiene” OR toothbrush* OR “tooth health”) | 3,559 |
| #5 | MAINSUBJECT.EXACT.EXPLODE("Oral Health") | 2,230 |
| #6 | #4 OR #5 | 3,559 |
| #7 | #3 AND #6 | 158 |

Controlled terms comment: Oral Health+ includes Dental Health

Scopus (Elsevier) n=5,155
Date of search: November 19, 2024
Search mode: Advanced Search

|  | **Query** | **Results** |
| --- | --- | --- |
| #1 | TITLE-ABS-KEY ( drama* OR game* OR play OR plays OR puppetry OR theater* OR theatre* OR storytelling OR "guided imagery" OR improvisation OR pantomime ) | 3,865,995 |
| #2 | TITLE-ABS-KEY ( "dental caries" OR "dental cavity" OR "dental health" OR "dental hygiene" OR "DMF index" OR "mouth health" OR "mouth hygiene" OR "oral health" OR "oral hygiene" OR toothbrush* OR "tooth health" ) | 166,999 |
| #3 | #1 AND #2 | 5,155 |

Web of Science Core Collection (Clarivate) n= 2,842
Indexes included: SCI-EXPANDED (1945-present), SSCI (1956-present), AHCI (1975-present), ESCI (2019-present)
Date of search: November 19, 2024
Search Mode: Exact search

|  | **Query** | **Results** |
| --- | --- | --- |
| #1 | TS=(drama* OR game* OR play OR plays OR puppetry OR theater* OR theatre* OR storytelling OR “guided imagery” OR improvisation OR pantomime ) | 2,728,131 |
| #2 | TS=(“dental caries” OR “dental cavity” OR “dental health” OR “dental hygiene” OR ”DMF index” OR ”mouth health” OR ”mouth hygiene” OR “oral health” OR “oral hygiene” OR toothbrush* OR “tooth health”) | 72,151 |
| #3 | #1 AND #2 | 2,842 |

## Results

| Total number of references: | 14,955 |
| --- | --- |
| Duplicates identified with Deduplicator, Institute for Evidence-Based Healthcare, Bond University, <https://sr-accelerator.com/#/deduplicator>  Focus: Relaxed | 7,704 |
| References transferred to Rayyan for TIAB screening | 7,251 |
